# Effect of Anthocyanin-Rich Tart Cherry Extract on Inflammatory Mediators and Adipokines Involved in Type 2 Diabetes in a High Fat Diet Induced Obesity Mouse Model

**DOI:** 10.3390/nu11091966

**Published:** 2019-08-21

**Authors:** Andrea Nemes, Judit Rita Homoki, Rita Kiss, Csaba Hegedűs, Diána Kovács, Barna Peitl, Ferenc Gál, László Stündl, Zoltán Szilvássy, Judit Remenyik

**Affiliations:** 1 Institute of Food Technology, University of Debrecen, H-4032 Debrecen, Hungary; 2 Institute of Pharmacology and Pharmacotherapy, Faculty of Medicine, University of Debrecen, H-4032 Debrecen, Hungary; 3 Cera-Med Ltd., H-4255 Debrecen, Hungary; 4 Pro–Recovery Kft., H-4032 Debrecen, Hungary

**Keywords:** sour cherry, anthocyanins, inflammatory mediators, adipokines, obesity, type 2 diabetes, mouse

## Abstract

Male C57BL/6J mice were used to determine the possible therapeutic effects of our previously described tart cherry extract in a chronic obesity mouse model on metabolic parameters, glucose tolerance, inflammatory mediators, and antioxidant capacity. The control group received standard mouse chow, and the high fat control group was switched to a high fat diet and tap water supplemented with 5% sucrose. The high fat + anthocyanin group received the high fat and sucrose diet, but received the anthocyanin-rich tart cherry extract dissolved in their drinking water. After six weeks, an oral glucose tolerance test was performed, and the water-soluble antioxidant capacity (ACW), superoxide dismutase (SOD) activity, and the plasma levels of insulin, C-peptide, leptin, IL-6, MCP-1, adiponectin and resistin were measured. The high fat diet increased body weight, reduced glucose tolerance, and caused an elevation in leptin, IL-6, MCP-1, and resistin levels. Furthermore, antioxidant capacity was decreased with a significant elevation of SOD activity. Anthocyanin treatment failed to reverse the effects of the high fat diet on body weight and glucose tolerance, but significantly reduced the leptin and IL-6 levels. The tart cherry extract also made a significant enhancement in antioxidant capacity and SOD activity. Our results show that chronic anthocyanin intake has a potential to enhance redox status and alleviate inflammation associated with obesity.

## 1. Introduction

### 1.1. Major Pathologic Features of Obesity and Type 2 Diabetes Mellitus (T2DM)

Obesity is one of the most widespread metabolic diseases and is also one of the biggest challenges for health care. Prevalence of obesity has increased threefold in the past decades, involving adults as well as children living not only in developed, but developing countries [1].

The World Health Organization considers obesity a fast-spreading global epidemic and emphasizes the importance of the prevalence or treatment of obesity-related cardiovascular or metabolic comorbidities. The most common complications of obesity are atherosclerosis, impaired glucose tolerance, insulin resistance and resulting T2DM, but it is also related to the increased risk of chronic diseases involving the respiratory system (sleep apnoea syndrome), the skeletal system, the endocrine system (menstrual disorders), or the gastrointestinal system (steatohepatitis, development of gallstones).

Recent research has revealed that adipose tissue plays an important role in regulating body glucose homeostasis. As a result of regular calorie overload, adipocyte dysfunction occurs, resulting in inflammatory responses. The inflammatory signal accelerates triglyceride metabolism and increases the levels of free fatty acids in the plasma. Consequently to prolonged free fatty acid excess, stress kinases are activated that inhibit insulin signaling pathways. The long-term elevation of circulating free fatty acids causes insulin resistance and β-cell damage [2].

The pathomechanism of T2DM is very complex and involves multiple peripheral and central mediators (insulin, leptin, ghrelin, GLP1), however, recent studies have underlined the importance of various adipocyte-derived peptides [3].

### 1.2. Role of the White Adipose Tissue (WAT) in the Patomechanism of T2DM

WAT plays a central role in energy homeostasis. The major function of WAT is to store and release energy, but it also has an important secretory function [4,5]. Adipokines, a class of proteins produced and secreted by WAT including several mediators that have some pro-inflammatory (leptin, interleukin-6, tumor necrosis factor α, resistin, MCP1, angiopoietin-like protein 2, chemerin, retinol binding protein 4) and anti-inflammatory properties (adiponectin, interleukin-10, visceral adipose tissue-derived serine protease inhibitor, omentin-1, apelin, secreted frizzled-related protein 5), are able to modulate whole body insulin sensitivity [6,7].

Obesity is accompanied by pathologically increased visceral fat mass that shows a tight correlation with the incidence of clinically important complications such as insulin resistance, T2DM, dyslipidemia, hypertonia, and metabolic syndrome, and contributes to decreased life expectancy. Studies have proven that in obesity, WAT increases the production and release of pro-inflammatory adipokines such as interleukin-6 (IL-6), monocyte chemotactic protein-1 (MCP-1) and tumor necrosis factor alpha (TNF-α), which contribute to the development of insulin resistance [8,9,10,11], while the expression and secretion of anti-inflammatory adiponectin decreases [11]. In addition, obesity is characterized by the infiltration of adipose tissue by mononuclear cells that also contribute to the production and secretion of inflammatory cytokines [12]. Obesity is often further characterized by the overproduction of reactive oxygen species (ROS) and the decrease of antioxidant capacity [13]. The central role of inflammation in the development of insulin resistance is further corroborated by studies that show that anti-inflammatory drugs are able to improve insulin sensitivity and related complications.

### 1.3. New Frontiers in the Prevention and Therapy of Obesity and T2DM

Unhealthy diet and altered eating habits, accompanied with sedentary lifestyle, genetic, and environmental factors contribute to the high prevalence of obesity and related complications, making it one of the most important civilization diseases. Diets rich in bioactive compounds can actively improve WAT function, therefore have beneficial effects on the clinical symptoms of obesity [14,15]. Despite recent developments in pharmacological strategies or surgical interventions, a reliable and safe therapy for obesity is still not at the expert’s disposal. In the last decade, there has been increasing scientific interest in investigating the possible therapeutic effects of bioactive compounds such as anthocyanins.

### 1.4. Characteristics of Anthocyanins

The most important challenge in nutrition science is the search for bioactive components from a natural origin that have a significant preventive effect. Anthocyanins have been at the center of nutrition science in recent years. Red fruits are known to synthesize these compounds in high concentrations; however, the anthocyanin profile is species-specific, so there are both qualitative and quantitative differences between species.

Cyanidin glycosides are predominantly present in Hungarian sour cherry varieties. The distinctive feature of cyanides is provided by their chemical structure, which determines their biological activity [16].

There have been numerous research studies on the biological effects of cyanidins: Homoki et al. [17] described that these compounds had high antioxidant activity and inhibited α-amylase activity; Akkarachiyasit et al. [18] published that cyanidins inhibited α-glucosidase activity. Furthermore, Jayaprakasam et al. [19] confirmed the effect of cyanidins to increase insulin secretion; Aguirre et al. [20] reported an improvement C Jun NH2-terminal kinase activity; Guo et al. [21] found that cyanidin-type anthocyanins increased the PPAR-α expression of hepatocytes in addition to increased expression of acyl-CoA oxidase and enzyme activity; and Changxing et al. [22] described a COX-1 inhibitor activity. The experimental data listed above suggest that cyanidins have blood glucose lowering and anti-inflammatory effects.

### 1.5. Aims

Dietary interventions can be adequate to treat obesity and prevent metabolic alterations. Therefore, the aim of the present study was to determine the possible therapeutic effects of our previously described tart cherry extract in a chronic obesity mouse model on metabolic parameters, glucose tolerance, inflammatory mediators, and antioxidant capacity. To assess the metabolic effects of the sour cherry extract, we investigated the glucose tolerance (fasting blood glucose and insulin response to oral glucose load), measured the levels of adipokines associated with body weight gain and insulin resistance such as leptin, IL-6, MCP-1, resistin, and adiponectin, and examined the antioxidant ability of the chronic sour cherry treatment by determining water-soluble antioxidant capacity (ACW) and superoxide dismutase (SOD) activity.

## 2. Materials and Methods

### 2.1. Fruit Samples

The Hungarian sour cherry “VN1” variety (selected from “Csengődi csokros”) was selected for the experiment based on its high antioxidant capacity as demonstrated by Homoki et al. [17]. The fruit samples that originated from the Research and Consulting Institute for Fruitgrowing, Újfehértó were collected between June and July of 2012, the period of time during which the cherries hold the highest anthocyanin concentration. Fruit samples were frozen immediately after picking, and were carried to the laboratory where they were kept in cold (−20 °C) dark storage [17].

### 2.2. Preparation of Athocyanins

The tart cherry samples were deseeded and homogenized with a Braun Multiquick mixer. The samples were extracted with an ethanol:water:acetic acid (25:24:1) mixture as a solvent. The extracts were mixed using a magnetic stirrer (MSH 300, IOSAN) for 1 h. After filtration, the samples were centrifuged (SIGMA 2-16 SARTORIUS) for 5 min at 10,000 rpm and further purified. For easier separation of the anthocyanins, a simple fractionation of the sour cherry extracts was performed using preconditioned Supelclean ENVI-18 SPE tubes [23]. The tubes were conditioned with 5 mL EtOH, then with 5 mL H_2_O, and finally 1 mL of fruit sample was applied. The anthocyanins were eluted with ethanol containing 20% water. The solvent was evaporated at 40 °C with a Heidolph rotary evaporator (Germany) [17].

### 2.3. UHPLC Analysis of the Anthocyanin-Rich Tart Cherry Extract

The UHPLC analysis was performed according to the procedure described by Nemes et al. [16]. Measurements were carried out using a CromasterUltraRs UHPLC, equipped with a diode array detector, automatic sampler, and Agilent OpenLAB software. The sample components were separated on a Phenomenex Kinetex column (2.6 μ, XB.C18, 100A, 100 × 4.6 mm). Eluent A was methanol and eluent B was 3% HCOOH (formic acid) in water. The following gradient elution program was used: 0 min solvent A 15%; 0–25 min solvent A to 30%; 25–30 min solvent A to 40%; and 30–40 min solvent A to 50%. The flow rate was 0.7 mL min−1 and the oven temperature was kept at 25 °C. The anthocyanin content was analyzed quantitatively by comparison with the corresponding authentic standards. UV–Vis detection was used at the 535 nm wavelength for anthocyanins. The appropriate amounts of sour cherry extracts were measured and dissolved in solvent A. The injection volume was 10 μL.

### 2.4. Ethics

All experiments involving mice were conducted in accordance with the European Community guiding principles for the care and use of experimental animals and the University of Debrecen Ethics Committee for Animal Research (ethical code number: 15/2013/DE MÁB, the date of approval of ethical submission: 15 April 2013).

Thirty five male C57BL/6J mice (obtained from Innovo Ltd. (Isaszeg, Hungary), the local distributor of The Jackson Laboratory) were used throughout the study.

### 2.5. Treatment of Animals

After arrival, the animals were habituated to the new environment for a week. Mice were housed in an animal room with 22–24 °C and 50–70% relative humidity. The lighting was set to 12 h light and 12 h dark period (lights off at 7 am). The animals received standard mouse chow (S8106-S011 SM R/M-Z+H; ssniff Spezialdiäten GmbH, Soest, Germany) and tap water ad libitum during the acclimatization period.

A week later, the animals were randomly divided into three groups. The control group (n = 11) received standard mouse chow and tap water ad libitum. The second group (n = 12) was switched to a high fat diet (RM AFE 45% FAT SY (P), Special Diets Services, United Kingdom) and tap water supplemented with 5% sucrose. The third group (n = 12) received a high fat diet and sucrose as the second group did, but also received anthocyanin-rich tart cherry extract in a daily dosage (*D*) of 60 mg/kg dissolved in their drinking water. The experimental protocol lasted for six weeks. The weight of the animals was registered twice a week, the water consumption was registered on a daily basis, and the anthocyanin solution was prepared accordingly to maintain the daily dosage protocol. The sucrose solution was freshly prepared every day, and the anthocyanin solution was prepared on a daily basis according to the daily water intake (*WI_d_*) and the body weight (*BW*) of the mice following the formula below:

(1) $$c\;\left(\mathrm{mg}/\mathrm{mL}\right)=\frac{D(\mathrm{mg}/\mathrm{kg})*\mathrm{BW}\left(\mathrm{kg}\right)}{{\mathrm{WI}}_{d}\;\left(\mathrm{mL}\right)}$$

### 2.6. Oral Glucose Tolerance Test (OGTT)

Glucose tolerance was determined by means of an oral glucose tolerance test (OGTT) in week 6 of the experimental period. After an overnight fast, the baseline blood glucose levels were measured (0 min), then the mice were given 2 g/kg glucose orally. Blood glucose levels were determined by means of a glucometer (Accu-Chek, Roche Diagnostics, Budaörs, Hungary) at 15, 30, 60, 90, and 120 min. Blood samples were collected by making a small incision in the lateral tail vein.

### 2.7. Blood Samples

After the OGTT, the mice were sacrificed and blood samples were taken. A total of 500 µL of whole, fresh blood was centrifuged for 10 min at 3000 rpm. Erythrocyte sediment (max 500 µL) was re-suspended three times in 1.5 mL normal saline solution and centrifuged again for 5 min at 3000 rpm each time before the supernatant was discarded. Lysis of the erythrocytes was achieved by adding 1 mL of cool water to the sediment and storing the sample at 4 °C in the dark for 15 min. Then, the sample was centrifuged for 5 min at 4 °C at 3000 rpm. The upper phase was frozen and stored at −70 °C for subsequent determinations.

### 2.8. Determination of Antioxidative Capacity in Water-Soluble Substances (ACW) by the Photochemiluminescence Method (PCL) Using the Photochem Instrument

The photochemiluminescence (PCL) method was carried out as described by Popov and Lewin [24]. In the PCL assay, the photochemical generation of free radicals is combined with sensitive detection by using chemiluminescence. The hydrophilic antioxidants of the plasma were measured in the Photochem with the ACW kit (Analytik Jena, Jena, Germany). A quantity of 10 µL prepared plasma, 1.5 mL reagent 1 (buffer solution pH 10.5), 1 mL reagent 2 (water), and 25 µL reagent 3 (photosensitizer) were mixed and measured. As these are standardized conditions, the results are comparable to other assays. The antioxidant potential was assayed by means of the lag phase.

### 2.9. Determination of the Antioxidant Capacity of the Enzyme SOD by PCL Method Using the Photochem Instrument

The antioxidative capacity of the enzyme SOD was measured with the ACW kit (Analytik Jena, Jena, Germany). Quantities of 10 µL prepared RBC, 1.5 mL reagent 1, 1 mL reagent 2, and 25 µL reagent 3 (photosensitizer) were mixed and measured. The SOD-enzyme (Superoxide Dismutase, Sigma Aldrich, Germany) was used as the standard [25].

### 2.10. Measurement of Plasma Adiponectin and Resistin Concentrations

Mouse adiponectin and resistin enzyme linked immunosorbent assay kits (MRP300 and MRSN00, R&D Systems, Minneapolis, USA) were used to measure the plasma concentration of the two proteins. Assays were performed according to the manufacturer’s instructions. All samples and standards were measured in duplicate. The plasma samples were diluted 5000 times in the case of adiponectin and 100 times in the case of resistin. Absorbance data were collected with a SPECTROstar Nano (BMG Labtech, Ortenberg, Germany) microplate reader set to 450 nm, then the raw ODs were corrected by the subtraction of absorbance measured at 540 nm. Sample concentrations were interpolated from a standard curve created by four parameter logistic curves fit using the MARS data analysis software 3.10 (BMG Labtech).

### 2.11. MILLIPLEX MAP Mouse Metabolic Hormone Magnetic Bead Panel

The plasma levels of insulin, C-peptide, leptin, interleukin-6 (IL-6), and monocyte chemotactic protein-1 (MCP-1) were determined by means of the MILLIPLEX MAP Mouse Metabolic Hormone Magnetic Bead Panel (MMHMAG-44K, EMD Millipore Corp., Billerica, MA, USA) according to the manufacturer’s instructions.

### 2.12. Statistics

The data distribution was analyzed with the D’Agostino-Pearson normality test. Data showing a Gaussian distribution were analyzed with one-way analysis of variance (ANOVA) followed by a modified *t*-test for repeated measures according to Tukey’s method. Data not showing the Gaussian distribution were analyzed with the nonparametric Kruskal–Wallis test. Data were presented as mean ± SEM.

## 3. Results

### 3.1. Composition of Anthocyanin-Rich Tart Cherry Extract

**Figure 1:**
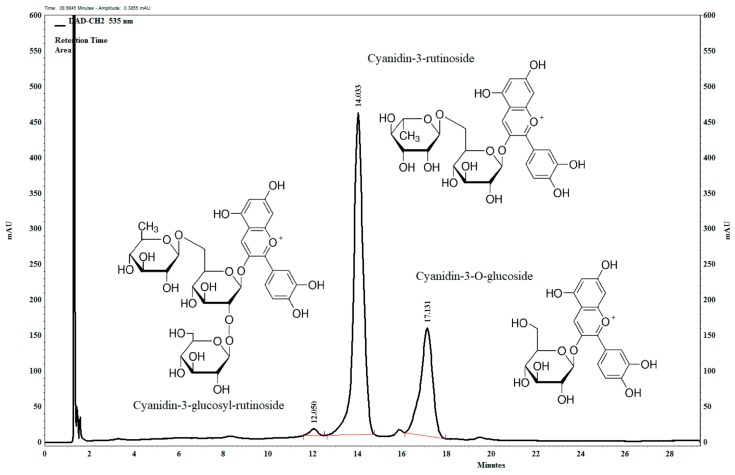
UHPLC chromatogram of the anthocyanin-rich tart cherry extract at 535 nm. Confirmed by the standard.

The main anthocyanin components in the anthocyanin-rich tart cherry extract (Figure 1) are the cyanidin-3-O-glucosyl-rutinoside, (1.15 mg/100 mg), cyanidin-3-O-rutinoside, (68.31 mg/100 mg), and cyanidin-3-O-glucoside (29.14 mg/100 mg).

The extract contains quercetin, quercetin-3-rutinoside, and apigenin (flavonoids) in small quantities (1–2%). These are precursor compounds in the biosynthesis of anthocyanins as well as other phenolic compounds like chlorogenic and caffeic acid in high concentrations in this fruit. The antioxidant activity of these compounds is also high [16,26].

### 3.2. Effect of Anthocyanin Treatment on Body Weight and Water Intake

**Figure 2:**
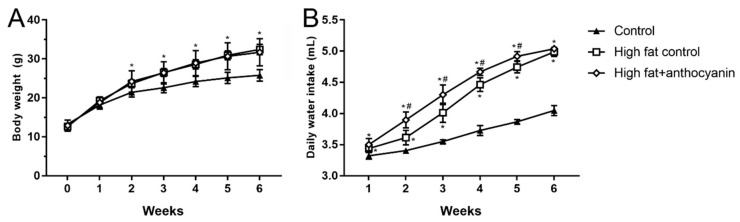
Effect of chronic anthocyanin treatment on body weight (Panel **A**) and daily water intake (Panel **B**). The * indicates a significant difference (*p* < 0.05) from the control group. The # indicates a significant difference (*p* < 0.05) between the high fat control and the high fat + anthocyanin groups.

At the beginning of the experimental period, the average body weight of the three groups did not show any significant difference between each other. However, the high fat control and the high fat + anthocyanin treated group showed a bigger daily increase in body weight when compared to the control group, and by week 2 of the experimental period, both groups grew into a significant difference when compared to the mice receiving the standard diet (Figure 2A). Anthocyanin treatment did not show any effect on body weight when compared to the high fat control group.

In average daily water intake, both the high fat control and the high fat + anthocyanin groups showed a statistically significant increase compared to the healthy control mice from week 1, and this difference was maintained throughout the whole experimental period (Figure 2B). From week 2, the anthocyanin treated animals showed a significantly elevated water consumption when compared to the high fat controls, and except for the last week, the difference was sustained between the two groups.

### 3.3. Effect of Anthocyanin Treatment on Glucose Tolerance

**Figure 3:**
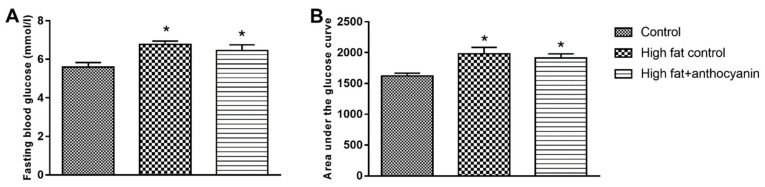
Effect of chronic anthocyanin treatment on the fasting blood glucose (Panel **A**) and area under the glucose curve (Panel **B**). The * indicates a significant difference (*p* < 0.05) from the control group.

At the end of the experimental period, the high fat control and the high fat + anthocyanin group showed a significant increase in the fasting blood glucose levels when compared to the control group (Figure 3A). After oral glucose administration, both the high fat control and the high fat + anthocyanin groups showed a decreased glucose tolerance when compared to the control mice, represented by a significantly higher area under the glucose curve (AUGU) (Figure 3B). Anthocyanin treatment induced no difference in the fasting plasma glucose and AUGC when compared to the high fat control mice.

### 3.4. Effect of Anthocyanin Treatment on Postprandial Levels of Insulin, C-Peptide, and Leptin

**Figure 4:**
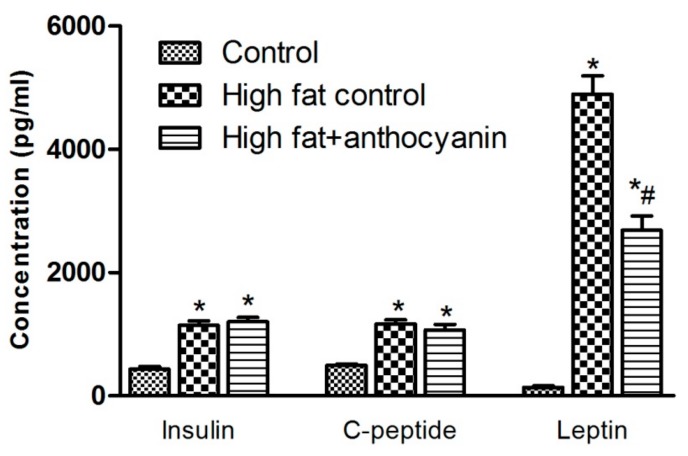
Effect of chronic anthocyanin treatment on postprandial levels of insulin, C-peptide and leptin. The * indicates significant difference (*p* < 0.05) from the control group. The # indicates a significant difference (*p* < 0.05) between the high fat control and the high fat + anthocyanin groups.

The oral glucose load in both the high fat control and high fat + anthocyanin group resulted in a significantly increased insulin response when compared to the control group (Figure 4), which was accompanied by a significant elevation of C-peptide levels 2 h after glucose administration, indicating a significant increase in insulin production in response to oral glucose challenge (Figure 4). Anthocyanin showed no effect in postprandial plasma insulin and C-peptide levels when compared to the high fat control. The high fat control group showed a more than 28-fold increase in postprandial leptin levels when compared to the control mice. On the other hand, though the high fat + anthocyanin group also showed a significant increase in leptin levels compared to the control group, anthocyanin also induced a significant decrease in leptin levels when compared to the high fat control mice (Figure 4).

### 3.5. Effect of Anthocyanin Treatment on IL-6 and MCP-1 Levels

**Figure 5:**
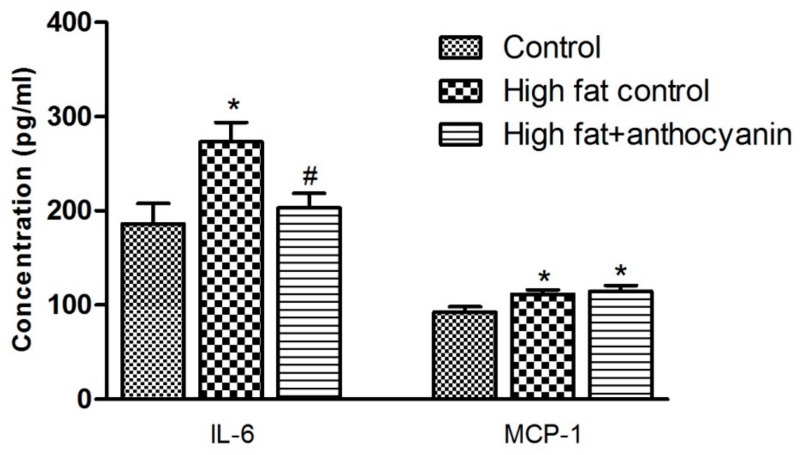
Effect of chronic anthocyanin treatment on the plasma levels of IL-6 and MCP-1. The * indicates a significant difference (*p* < 0.05) from the control group. The # indicates a significant difference (*p* < 0.05) between the high fat control and the high fat + anthocyanin groups.

At the end of the experimental period, the IL-6 levels were significantly increased in the high fat control group when compared to the healthy control mice, however, in the high fat + anthocyanin group, the IL-6 levels showed no difference when compared to the control animals but were significantly lower than that in the high fat controls (Figure 5). Levels of MCP-1 were significantly elevated in both the high fat control and high fat + anthocyanin groups when compared to the controls (Figure 5).

### 3.6. Effect of Anthocyanin Treatment on water-soluble antioxidant capacity (ACW)

**Figure 6:**
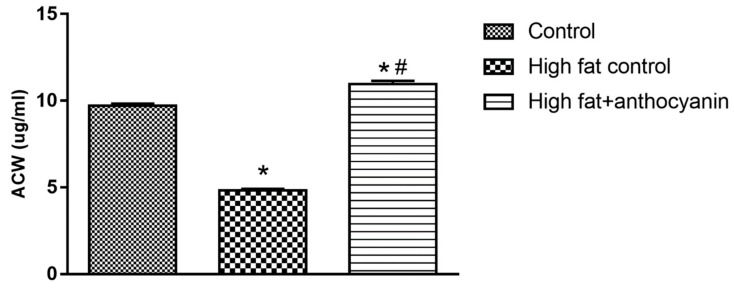
Effect of chronic anthocyanin treatment on water-soluble antioxidant capacity (ACW) in the blood. The * indicates a significant difference (*p* < 0.05) from the control group. The # indicates a significant difference (*p* < 0.05) between the high fat control and the high fat + anthocyanin groups.

A high fat diet significantly decreased the ACW levels, as demonstrated by a significant difference between the healthy control and high fat control group. Anthocyanin treatment successfully prevented this decrease; furthermore, the high fat + anthocyanin mice even showed a slight, but significant increase in the ACW levels when compared to the healthy controls (Figure 6).

### 3.7. Effect of Anthocyanin Treatment on Superoxide Dismutase (SOD)

**Figure 7:**
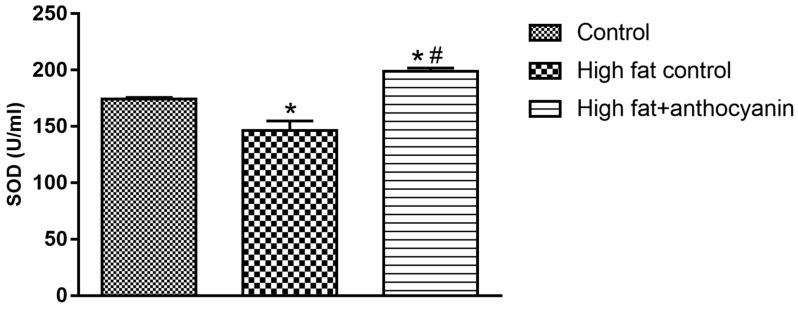
Effect of chronic anthocyanin treatment on the plasma levels of SOD. The * indicates a significant difference (*p* < 0.05) from the control group. The # indicates a significant difference (*p* < 0.05) between the high fat control and the high fat + anthocyanin groups.

In the high fat control mice, the levels of SOD decreased significantly by the end of the experimental period. Anthocyanin treatment effectively elevated SOD when compared to both the healthy and high fat controls (Figure 7).

### 3.8. Effect of Anthocyanin Treatment on Resistin and Adiponectin

**Figure 8:**
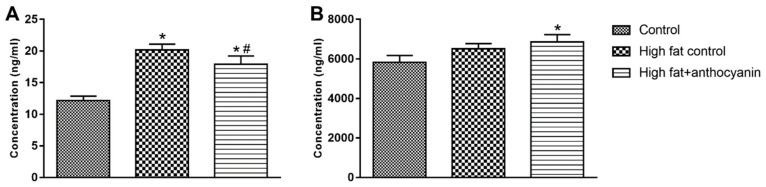
Effect of chronic anthocyanin treatment on the plasma levels of resistin (Panel **A**) and adiponectin (Panel **B**). The * indicates a significant difference (*p* < 0.05) from the control group. The # indicates a significant difference (*p* < 0.05) between the high fat control and the high fat + anthocyanin groups.

The concentration of resistin in the plasma was significantly increased in the high fat control group, but the anthocyanin treatment effectively decreased it (Figure 8A). The concentration of adiponectin was not significantly altered by the high fat diet, but the anthocyanin treatment significantly increased adiponectin when compared to the control group (Figure 8B).

## 4. Discussion

**Figure 9:**
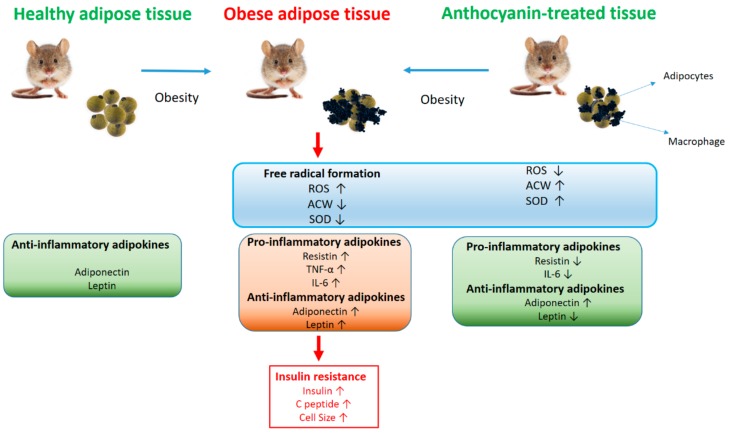
Overview of the results. Graphical interpretation of the result show the modification of the followed main investigations: oxidative stress parameters, cytokines, and adipokines in healthy, obese, and obese anthocyanin treated animal models.

The main results of our study are briefly summarized in Figure 9. In our in vivo experiment, the high fat diet significantly increased body weight in the six week experimental period. The combination of high fat diet and drinking water supplemented with sucrose was effective in developing obesity and insulin resistance [27]. There is a variety of anthocyanin treatment options in the mouse obesity models described in the literature where different berries, peel powders, berry juice, or purified anthocyanins have been given as an intervention, and the doses applied also varied. Most animal studies have reported beneficial results after chronic anthocyanin treatment in the dose range between 40 and 80 mg/kg, and studies with obese humans have reported comparable results in the dose range translated from those used in mouse experiments [28,29]. We chose the 60 mg/kg anthocyanin dose accordingly. Chronic oral anthocyanin treatment did not protect the animals from diet induced weight gain, which is in accordance with the results of other laboratories [30,31].

The obesity model with a high fat diet and tap water supplemented with sucrose was effective in causing fasting hyperglycemia, a condition often seen in obesity and type 2 diabetes. Chronic anthocyanin treatment was unable to significantly decrease fasting hyperglycemia compared to the obese control mice, and our findings are corroborated by the results of other groups [27,31,32].

By the end of the experimental period, a high fat diet and sucrose water resulted in a marked decrease of glucose tolerance in both the obese control and the anthocyanin treated groups. The significantly increased insulin response accompanied by an elevated C-peptide secretion in response to the oral glucose load was unable to counteract the blood glucose changes comparable to healthy controls. A transient increase in fatty acid levels can also be considered as physiological stimulation of insulin production as insulin secretion may temporarily increase to maintain metabolic balance. However, a prolonged fatty acid overload impairs β cell function. In our experiment, we found that hypercaloric diet increased insulin secretion. The anthocyanin treatment failed to counteract the high fat diet effect on insulin secretion. Cyanidin-3-o-β glycoside among the anthocyanins has been shown to be able to increase the insulin sensitivity of the cells by inactivating JNK stress kinase or not converting serine insulin receptor substrate-1 [20]. Compared to the results of other groups, we found conflicts regarding anthocyanin treatment and glucose tolerance. Several reports have found an improvement in glucose tolerance [33,34,35], while other groups reported no change in fasting hyperglycemia and glucose tolerance by anthocyanin treatment in obese rodent models [36,37]. A possible explanation to solve these controversial reports could be that the majority of investigations have evaluated the metabolic effects of fruit juice or extracts made from native plants rich in anthocyanins. It has been proven that different fruits, vegetables, and plants contain various anthocyanins and other possibly bioactive compounds in different concentrations and proportions [38], which can in turn act differently in animal models. The important role of anthocyanin concentration was highlighted by Oyama et al. [36], who reported that 0.5% juçara pulp supplementation significantly improved glucose tolerance in a high calorie mouse model. On the other hand, the 2% supplementation was unable to decrease the area under the glucose curve after an oral glucose load compared to the obese controls [36].

It is well established that white adipose tissue not only acts as an energy storage, but also plays an important endocrine function. Hormones produced by adipose tissue, referred to as adipokines, are secreted into the blood stream during physiological conditions, but weight gain and obesity pathologically change the gene expression and secretory pattern of such compounds, significantly elevating the levels of proinflammatory adipokines such as leptin, MCP-1, IL-6, and resistin while decreasing the production of anti-inflammatory adiponectin and IL-10. Such changes contribute to the development of tissue inflammation, insulin resistance, and other symptoms of metabolic syndrome.

The first discovered and described adipokine, leptin, is known as the “satiety hormone” or “starvation hormone” as it inhibits hunger and regulates body weight and energy homeostasis. Leptin is produced by adipocytes and leptin levels are proportional to the amount of body fat [39]. It also regulates appetite by acting on receptors in the arcuate nucleus of the hypothalamus. In obesity, leptin levels are increased, but the activity of leptin is decreased due to leptin resistance [40]. Elevated leptin levels also contribute to insulin resistance and show a positive correlation to the risk of cardiovascular diseases and metabolic syndrome [41]. Furthermore, leptin is able to modulate immune reactions [42]. In our study, a high fat diet effectively increased the leptin levels in both the high fat control and high fat + anthocyanin treated mice. High fat fed mice showed increased body weight and resulting hyperleptinemia, which could contribute to glucose intolerance. Chronic anthocyanin treatment significantly decreased leptin level when compared to the high fat control mice, though it was still significantly high when compared to the healthy controls. Thus, it is not surprising that anthocyanin treatment did not significantly improve glucose tolerance nor did it decrease body weight by the end of the experimental period. Other reports, but not all, indicate that anthocyanin is able to decrease leptin levels and body weight in 12 weeks, which in turn is accompanied by the improvement of fasting insulin sensitivity [32,43,44]. We cannot dismiss the possibility that our tart cherry extract in bigger doses, or with a longer treatment period would be able to cause a significant improvement in glucose metabolism, but answering this question will require further experiments.

The analysis of the additional proinflammatory adipokines showed that a high fat diet elevated the levels of MCP-1, IL-6, and resistin, and our results were corroborated by reports from other laboratories [30,45,46]. MCP-1 is an adipokine released by fat cells. The most important role of MCP-1 is to regulate the migration and infiltration of monocytes and macrophages, but it can also contribute to insulin resistance, acting together with other mediators such as IL-6 and TNF-α [47]. IL-6 is a multifunctional cytokine that plays an important role in inflammation and autoimmune disorders including diabetes, atherosclerosis, rheumatoid arthritis, systemic lupus erythematosus, or psoriasis. In obesity, IL-6 is released from the adipocytes and deteriorates insulin sensitivity of the tissues [48]. Resistin is a peptide hormone that is produced by mature adipocytes in mice [49] and macrophages and monocytes in humans [50]. Proinflammatory cytokines such as IL-6 and TNF-α are able to induce resistin expression [51], while the insulin sensitizing rosiglitazone, a PPARγ agonist, has an inhibitory effect on resistin expression in WAT [52]. Resistin promotes the expression of IL-6 and TNF-α in human monocyte cells [53] and is able to directly inhibit the anti-inflammatory effects of adiponectin [54]. The expression and secretion of MCP-1, IL-6, and resistin are increased in obesity, playing an important role in the appearance of the inflammatory M1 type macrophages, and decreasing the expression and production of the protective adiponectin and IL-10 in the adipose tissue [11]. They are also able to inhibit insulin signaling [55], which was also supported by our glucose tolerance results that showed a marked insulin resistance in the mice kept on a high fat diet. The sour cherry extract significantly decreased the IL-6 levels, which was comparable to the results of other groups [56,57], but it is worth mentioning that there are reports where anthocyanin treatment did not prove to be effective in decreasing IL-6 levels in a diet induced obesity model [30,45]. According to our results, the sour cherry extract was unable to decrease MCP-1 levels. In different laboratories, anthocyanin treatment significantly decreased MCP-1, but it is important to note that in DeFuria’s study, anthocyanin was unable to decrease the elevated MCP-1 mRNA expression related to the diet induced obesity model [31]. Adiponectin is an anti-inflammatory adipokine. Expressed and secreted mainly from adipocytes, adiponectin shows an inverse correlation with the risk of obesity and insulin resistance [58]. Adipokines promoting inflammation such as IL-6 and TNF-α effectively decrease adiponectin expression as well as oxidative stress [59]. The PPARγ agonist rosiglitazone stimulates adiponectin secretion [60,61]. Evidence suggests that adiponectin is able to decrease TNF expression [62], stimulate the production of anti-inflammatory interleukin IL-10 [63], and is able to switch the profile of macrophages from proinflammatory M1-type to the anti-inflammatory M2-type [64].

Tsuda described that anthocyanins increased the secretion of adiponectin [65]. This was confirmed by our study as the anthocyanins of sour cherry extract enhanced the separation of adiponectin in animals that were fed with a high fat diet.

In our experiment, the high fat control mice showed significant differences when compared to the healthy controls, but we must point out that it is difficult to compare the studies in every detail as there are substantial differences in the literature regarding the obesity or insulin resistance model, rodent strain, and the chemical composition, dosage, and treatment duration of the various compounds and extracts used. We can assume that the above inconsistencies induced differences in the adipokine gene expression patterns, which could explain why the adiponectin levels did not change either in the obesity model or in response to the anthocyanin treatment. However, we underline that chronic treatment with tart cherry extract decreased leptin and IL-6 levels, which demonstrates that the applied dose of anthocyanin has an anti-inflammatory potential, and we can assume that a higher dose of anthocyanin and/or a longer period of treatment may effectively improve insulin sensitivity in the diet induced obese model, but demonstrating our hypothesis will require further experiments.

The chemical structure of the anthocyanins determines their pharmacological activity. The antioxidant activity of these compounds depends on the level of hydroxylation on the 3′ and 4′ positions of the B-ring structure. This structure is also a pivotal factor of their radical scavenging activity, so anthocyanins are effective donors of hydrogen [16,66]. Their antioxidant effect is realized in two ways. Primarily, they can increase the activity of redox homeostasis enzymes, like catalase (CAT), glutathione peroxidase (GPx), and glutathione reductase (GR) [67]. Furthermore, they can increase the expression of the gene encoding the γ-glutamylcysteine synthetase catalytic subunit, a protein reported to be the rate-limiting step in glutathione (GSH) synthesis [68]. Therefore, anthocyanins can enhance glutathione concentration, and SOD concentration also increases. Mulabagal et al. demonstrated that cyanidin glycosides could inhibit the lipid peroxidation (LPO) in vitro [69]. Furthermore, Nizamutdinova et al. investigated the effect of cyanidin-glucosides in streptozotocin (STZ)-induced diabetic rats, and found that these compounds can increase SOD activity [70]. Due to dietary obesity, the concentration of adipocytokines (produced by adipocytes) increases. This correlates with the degree of oxidative stress, and thanks to oxidative stress, H_2_O_2_ production increases [71]. H_2_O_2_ inactivates SOD, resulting in reduced activity. However, anthocyanins can directly increase SOD activity [67]. In accordance with other literature, we found data that anthocyanins increased SOD activity, nonetheless the effect was more significant in comparison with the HFD control. Fatty acids are transported across the cell membrane and link to the cell metabolism as fatty-acyl-CoA, which enters the mitochondria and peroxisome by carnitine transport to be degraded to acetyl-CoA during the β-oxidation process. However, this mechanism enhances the production of reactive oxygen species (ROS). In cases such as obesity, free fatty acid levels are elevated in the blood, in addition, pro-oxidants are already present at extremely high concentrations due to inflammatory processes, thereby aggravating oxidative stress. Simultaneously, palmitate-induced superoxide production in muscle cells causes β-oxidation, but NADPH oxidase is also activated [72]. ROS accumulation increases the permeability of the mitochondrial membrane, resulting in further prooxidant production [73]. Thus, lipotoxicity is actually based on oxidative stress. Free fatty acids are physiologically transported in the blood by serum albumin. However, if circulating free fatty acid levels are increased, albumin binding sites can become saturated, resulting in profound conformational changes, which modifies the redox state of the Cys-34-SH group responsible for the antioxidant capacity of albumin [74]. Our measurements prove that a fat-rich diet impairs the antioxidant activity of the plasma. Chronic anthocyanin treatment was able to counteract the decrease of antioxidant capacity seen in our obesity model, and a slight, but significant increase was also observed when compared to the healthy controls.

## 5. Conclusions

Hypercaloric food intake in the long term results in the dysfunction of adipose tissue, which plays a major role in a number of disorders. The cytokine profile secreted by the adipose tissue changes, thus the altered secretion of adipocyte hormones leads to obesity-induced insulin resistance, inflammation, changes in the antioxidant-prooxidant balance, and decreased plasma antioxidant capacity. The evolved local inflammation accelerates the triglyceride metabolism and significantly increases free fatty acid levels in the plasma. On one hand, this results in a reduction of plasma antioxidant capacity. Additionally, the degradation of fatty acids during β-oxidation results in significant prooxidant production. Furthermore, increased levels of ROS induces the synthesis of factors that results in the production of various inflammatory cytokines, thus promoting the development of insulin resistance, which later leads to β cell depletion and type 1 diabetes.

The anthocyanins of sour cherry (cyanidin-3-O-glucoside, cyanidin-3-O-glucosyl-rutinoside, and cyanidin-3-O-rutinoside) can effectively improve the proinflammatory cytokine profile of adipocytes and the antioxidant status in the body, and thus might have a preventive effect on the development of metabolic syndrome. Our results confirm that anthocyanin-based therapy can be a promising alternative to rebalance the systemic metabolic homeostasis shifted by obesity.
